# Global research trends on anti-PD-1/anti-PD-L1 immunotherapy for triple-negative breast cancer: A scientometric analysis

**DOI:** 10.3389/fonc.2022.1002667

**Published:** 2023-01-11

**Authors:** Jinyao Wu, Yaokun Chen, Lingzhi Chen, Zeqi Ji, Huiting Tian, Daitian Zheng, Qiuping Yang, Yiyuan Liu, Jiehui Cai, Jiehua Zheng, Yexi Chen, Zhiyang Li

**Affiliations:** Department of Thyroid, Breast and Hernia Surgery, The Second Affiliated Hospital of Shantou University Medical College, Shantou, Guangdong, China

**Keywords:** triple-negative breast cancer, PD-L1, PD-1, immunotherapy, scientometrics, VOSviewer, citespace, bibliometrix

## Abstract

In recent years, anti-PD-1/anti-PD-L1 has been considered to be a valuable therapeutic target and prognostic indicator for triple-negative breast cancer. We analyzed all publications published in the field from their inception until the present day in order to determine the current research status and hotspots. All related publications were searched on the Web of Science. Our research used R-studio (bibliometrix package), VOSviewer, and CiteSpace to analyze and obtain annual publications and citation information, articles, highest publication countries and affiliations, influential journals and authors, keyword analysis, and keyword bursts. In total, 851 documents were retrieved including 628 articles and 223 review articles. The output of publications increased year by year from 2013 to 2021. However, the average article citation times reached the top in 2014 but generally showed a downward trend from 2014 to 2021. It was an article written by Schmid et al. in 2018 that received the most citations. With regard to publications, citations, and link strength, among the top countries was the United States. Cancers was the most published journal. Schmid and Loi ranked top in total citations and h-index. Schmid has the largest M-index and Loi has the most publication. The keywords that received the most attention were “Immunotherapy”, “PD-L1”, “Triple-negative breast cancer”, “Tumor-infiltrating lymphocytes”, and “Expression”. According to the report, this current research focuses on immunotherapy for triple-negative breast cancer and the expression of PD-L1 and tumor-infiltrating lymphocytes (TILs). Pembrolizumab and Atezolizumab plus chemotherapy have completed the Phase 3 clinical trial. However, the biomarkers were limited in predicting the treatment prognosis. Through the scientometric analysis, we can understand the current research status and potential research points in this filed and provide research direction for researchers.

## 1 Introduction

In 2020, breast cancer (BC) overtook lung cancer in women as the most frequently diagnosed cancer, with an estimated 2.3 million new cases (11.7%) and 684,996 deaths (6.9%). Female breast cancer mortality rates in developing countries were much higher than in developed countries (1). Traditionally, BC has been classified based on the functional expression of steroid hormone receptors, including estrogen receptor (ER), progesterone receptor (PR), and epidermal growth factor receptor 2 (HER2) (2). Triple-negative breast cancer (TNBC), the absence of expression of ER, PR, and HER2, is an extremely aggressive and heterogeneous tumor, accounting for 15%–20% of all breast cancer cases (3). Surgical resection, radiation, and general chemotherapy remain the main way to treat such patients (3). This classic treatment has limitations, faces serious tumor recurrence and multidrug resistance (MDR) problems, and lacks precisely targeted therapy (4).

TNBC’s molecular analysis has identified potential opportunities for targeted intervention, including advanced chemotherapy techniques to respond to DNA damage, angiogenic inhibitors, immunodeficiency inhibitors, and even antiandrogenic hormones (5). Modulating the immune system may be the better way to treat TNBC, which progresses due to complex interactions with the immunity system (3). Compare with other types of cancer, breast cancer has a lower number of tumor-infiltrating immune system cells with tumor-rejecting capacity (primarily T lymphocytes) (6). A high level of PD-L1 correlates with high-risk scores such as tumor multiplication, tumor size, aggressive molecular subtype, ER-negative, and lymph node metastases in breast cancer patients (7, 8). Compared with other subtypes of breast cancer, TNBC has stronger infiltration immunity, genome instability, higher positivity to express PD-L1, and more non-synonymous mutations (9).

Nowadays, the combination of atezolizumab and nab-paclitaxel has been approved for patients with unresectable locally advanced or metastatic tumors expressing PD-L1 (10). Some studies have found that immune checkpoint inhibitor (ICI) as the first-line treatment has been superior to follow-up treatment for advanced or metastatic TNBC. Pembrolizumab plus chemotherapy has shown good efficacy as neoadjuvant therapy in early TNBC (11).

Despite their considerable success, there are still many challenges to overcome with immune checkpoint inhibitors. Some patients remain unresponsive to PD-1 blockade, and some suffer resistance or treatment-related side effects (12). To understand the current studies on hot points and potential research points in this field, we carried out a scientometric analysis related to anti-PD-1/anti-PD-L1 immunotherapy for TNBC.

We conducted this scientometric analysis to obtain information about the number of annual publications and citations, countries and organizations, journals and research categories, authors, references, and keywords in anti-PD-1/anti-PD-L1 immunotherapy for TNBC. The purpose was to show the current status of research and potential research points in this field and provide research direction for researchers.

## 2 Materials and methods

### 2.1 Data collection

We searched all publications related to anti-PD-1/anti-PD-L1 immunotherapy and TNBC from the Web of Science Core Collection Database including editions of Science citation index expanded (SCI-Expanded) 2003 to present. The keywords of the search strategies included “anti-PD 1”, “anti-PD L1” “Triple-Negative Breast Cancer”, and “Immune Checkpoint Inhibitor”. This research was conducted on July 11, 2022, and we selected the publications from the first published to the present. Total results of 1286 documents were produced. By restricting our search to scholarly articles or reviews and English as the language, we found 851 publications, including 628 scholarly articles and 223 reviews. There were 435 publications excluded, including 362 meeting abstracts, 36 editorial materials, 14 corrections, 12 letters, 6 news items, 2 non-English articles, 2 non-English review articles, and 1 withdrawal item. At last, the number of publications performed that bibliometric analysis was 851 including 628 articles (73.80%) and 223 review articles (26.20%).

### 2.2 Analysis

We extracted information by using the Web of Science’s “analyze results” function, including publication years, document types, authors, affiliations, publication titles, publishers, research areas, and countries/regions. The “citation report creation” function of WoS was used to obtain the number of citing article, the sum of times cited, and the average citations per item (ACI).

Bibliometrix is an R-package for bibliometric quantitative research that provides a set of tools. Biblioshiny provides bibliometrix with a cleaner and more convenient interface, with multiple effective statistical algorithms, high-quality numerical routines, and data visualization (13). We performed a complete scientometric analysis by using R-studio’s bibliometrix package (version 4.2.0) and imported the retrieved data files into Biblioshiny to obtain the main information of publications, including the time span, annual scientific output, average citations per year, the trend of authors, the impact of source and authors, and so on.

In addition, we imported the data files collected from WoS into the VOSviewer software (version 1.6.18). VOSviewer is a free computer program for bibliometric analysis and generating visualization maps. Its capabilities are particularly useful for displaying large bibliometrics in an easy-to-understand way (14). We used VOSviewer to obtain the network and overlay map about co-authorship of different countries and organizations, co-citation analysis of authors and references, co-occurrence analysis of keywords, etc.

CiteSpace (version 6.1.R2) citation bursts can assign two attributes: the intensity of the burst and the duration of the burst state (15). We use CiteSpace to analyze citation bursts of keyword.

Statistical analyses were conducted with Stata MP17. We perform linear exponential curve fitting analysis on annual scientific production. Correlation and statistical significance were verified using the Spearman correlation coefficient. A p-value smaller than 0.05 was considered statistically significant.

**Figure 1:**
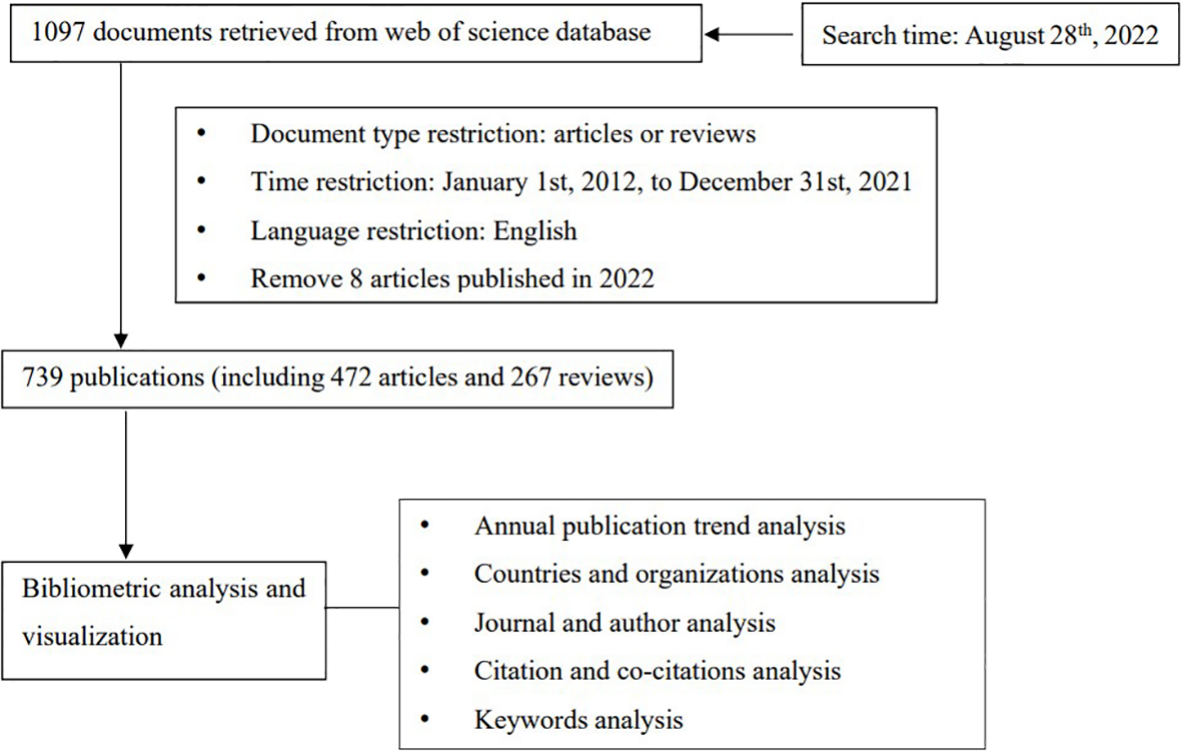
The process of literature search and analysis.

The steps of collecting and analyzing are shown in **Figure 1**.

## 3 Results

### 3.1 Growth and trend of publications and citations

**Figure 2:**
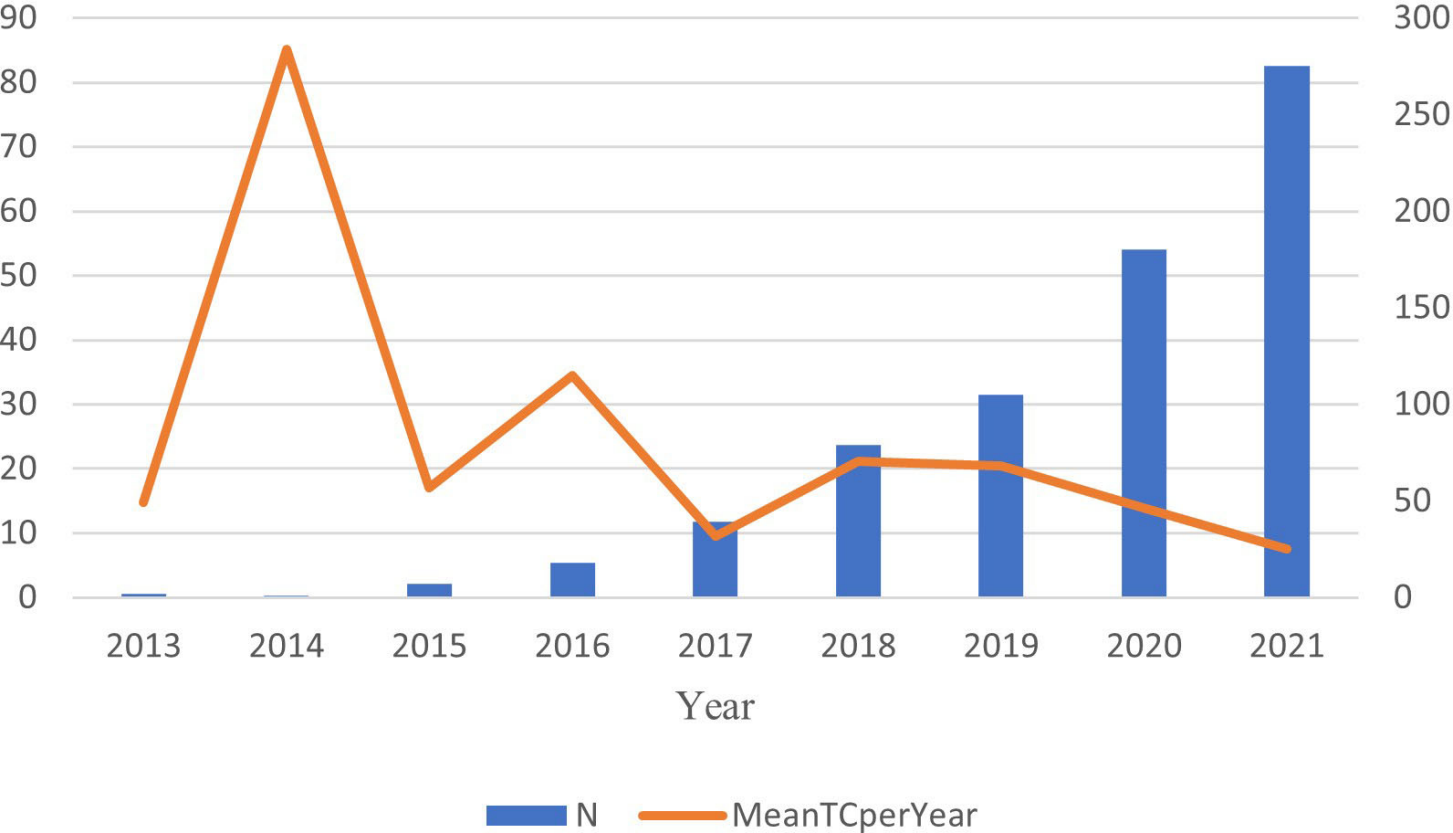
The annual scientific production and average citations per year.

A total of 851 documents related to this subject were retrieved from the Web of Science (WoS). Since 2022 is not yet over, we analyze growth trends from the emergence of the field to 2021. From two documents published in 2013 (0.24%) to 280 documents (32.90%) published in 2021, there was an upward trend (**Figure 2**) (r^2^ = 0.9257 [CI: 0.836–1.03]; p < 0.001]). The output of publications increased year by year from 2013 to 2021 and exceeded 100 in 2019, presenting a strong overall growth trend (annual growth rate: 59.28%).

Citation analysis is a simple, objective method of assessing the quality of published articles, journals, research organizations, and even individual researchers. The times of articles cited can reflect the scientific impact (16). Based on **Figure 2**, this field first appeared in 2013. The average article citation times generally showed a downward trend from 2014 to 2021. In 2014, the average number of citations reached the peak (85.1) and then gradually showed a fluctuation and decline, and the average number of citations in 2021 reached 7.5. Based on the citation report function of WoS, 16.037 citing documents included 761 self-citations and 15,276 without self-citation (accounted for 95.25%). There were 27.646 cited times, of which 23,041 were without self-cited, accounting for 83.34%. The average number of citations was 32.49 times.

### 3.2 Country and organization analysis

A total number of 851 documents contributed to this field of research by 65 countries and regions, including both developed and developing nations. **Supplementary Table 1** shows the top 10 producing countries and cp-cited countries. Documents from the United States accounted for the majority (n = 348, 40.89%), followed by documents from China (n = 227, 26.68%) and Italy (n = 76, 8.93%). There were the most citations in publications published in the United States (n = 11906), England ranked second (n = 3350), followed by China (n = 3060), Italy (n = 2099), and Germany (n = 1158). Both in terms of publication and citation numbers, the United States had the most. A total of 1586 organizations contributed to this research field. According to **Supplementary Table 1**, among the top 10 organizations with publications, Harvard university was the most productive affiliation (n = 55, accounted for 6.46% of all). It was followed by University of Texas System (n = 44, 5.17%), and Dana Farber Cancer Institute (n = 43, 5.05%). There are six organizations from the United States (60%).

**Figure 3:**
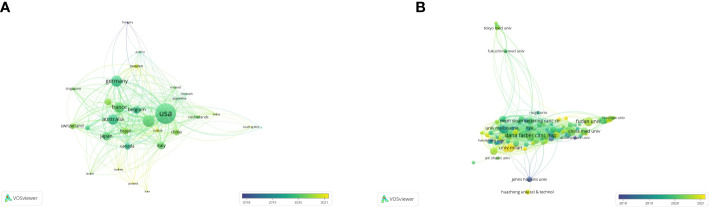
The co-authorship analysis of countries and organizations. **(A)** The overlay map of co-authorship between countries (blue: earlier, yellow: later). **(B)** The overlay map of co-authorship between organizations (blue: earlier, yellow: later).

According to the co-authorship analysis, a total of 31 countries published at least five papers in this research (**Figure 3A**). Ranked by total link strength, the United States was at the top (total link strength = 352), followed by England (201), Germany (184), Australia (175), and France (172). The circle size represents the strength of a country’s connections with other countries, and the thickness of lines and distance between two countries indicates their relatedness. The more yellow the color in the overlay visualization, the closer its average active time is to the present. Developed countries produced more publications and cited articles than developing countries. Recently, some developing countries like China (total link strength = 103) and Sweden (63) began to rise in the world in this field.

By analyzing the co-authorship of organizations, 125 organizations published at least five papers, and only 121 were connected (**Figure 3B**). Based on the total link strength, Dana Farmer Cancer Inst was the strongest (177), followed by University of California, San Francisco (144), Queen Mary University of London (133), Genentech Inc. (113), and Aichi Cancer Center Hospital (110). Recently, the University of Milan (68) and IRCCS European Institute of Oncology (55) began to rise in the world in this field.

### 3.3 Journal and research category analysis

In total, these 851 documents were published in 280 different journals. **Table 1** shows the top 10 most influential journals in this field. A total of 173 papers were published in these top 10 journals, accounting for 20.33% of all publications. Cancers were the most active journal which contributed 35 publications, followed by Frontiers in Oncology which had 25 publications, Journal for Immunotherapy of Cancer (n = 20), Clinical Cancer Research (n = 18), and Annals of Oncology (n = 15). In the cited source, New England Journal of Medicine had the most citations at 2444, followed by Annals of Oncology (1725), Journal of Clinical Oncology (1314), Clinical Cancer Research (1249), and Nature Reviews Clinical Oncology (1202). According to the h-index, Annals of Oncology had the largest h-index of 14, followed by Clinical Cancer Research (h-index = 12), Journal for Immunotherapy of Cancer (h-index = 10), Oncotarget (h-index = 10), and Cancer Research (h-index = 9). Annals of Oncology was the journal with the most citations and h-index. These top 10 journals were published from 2013 to 2019.

**Table 1:** Top 10 most influential journals.

Journal (country)	h-index	g-index	m-index	TC	NP	PY start
Annals of Oncology (UK)	14	15	2.8	1725	15	2018
Clinical Cancer Research (USA)	12	18	1.2	1249	18	2013
Journal for Immunotherapy of Cancer (UK)	10	20	2.5	618	20	2019
Oncotarget (USA)	10	12	1.25	647	12	2015
Cancer Research (USA)	9	11	1.285714286	476	11	2016
Cancers (Switzerland)	9	21	2.25	493	35	2019
Oncoimmunology (USA)	9	13	1.285714286	217	13	2016
Breast Cancer Research (UK)	8	10	1	415	10	2015
Breast Cancer Research and Treatment (USA)	8	14	1.333333333	474	14	2017
BMC Cancer (UK)	6	10	1	224	10	2017

The source growth was shown in **Supplementary Figure 2**. Annals of Oncology surpassed Breast Cancer Research in 2016 to become the most published journal until the end of 2019. Cancer successfully overtook Annals of Oncology as the most published journal in 2020 and has continued up to now. Cancers and Frontiers in Oncology maintain a good number of publications output in recent years.

There were 52 research categories and 63 publishers. According to the number of publications, the top 10 subject categories and publishers were lined in **Supplementary Table 2**. “Oncology” accounted for more than half of all documents (514, 60.40%), followed by “Immunology” (83, 9.75%) and “Pharmacology Pharmacy” (79, 9.28%). As for the publisher, “Springer Nature” published the most number of publications (156, 18.33%), followed by “Elsevier” (130, 15.28%) and “Mdpi” (89, 10.46%).

### 3.4 Author analysis

These 851 publications were contributed by 5251 authors, including 14 authors of single-authored documents and 5237 authors of multi-authored documents. There were 16 documents written individually. There was an average of 6.17 authors per document and 8.72 co-authors per document. **Table 2** shows the top 10 published authors with highest impact in this field. Loi was the author with the most contributions in this field, with 22 published articles accounting for 3.88%, followed by Li with 18 publications (2.12%) and Wang (n = 18, 2.12%); 29 authors have authored over 10 articles, accounting for 3.41%. The top 10 authors published in 2013 to 1019. In the cited author, Schmid had the most citations of 4806, followed by Loi (4322) and Iwata (3910). According to the h-index ranking, the highest h-index of Loi was 18, followed by Schmid (h-index = 14) and Adams (h-index = 11). There were nine authors whose h-index was higher than 10. The m-index is equal to the h-index divided by the number of years since the scientist first was active, to eliminate the bias caused by different seniority (17). Schmid had the highest m-index of 2.800, followed by Li (m-index=2.250) and Adams S (m-index = 2.200). According to h-index, m-index, total citations, and contributions, Schmid is the author with the highest academic level in this field, while Loi is the author with the most output and contributions.

**Table 2:** Top 10 most influential authors.

Author	h-index	g-index	m-index	TC	NP	PY start
Loi S	18	22	1.8	4322	22	2013
Schmid P	14	17	2.8	4806	17	2018
Adams S	11	12	2.2	3625	12	2018
Emens La	11	15	1.375	2819	15	2015
Pusztai L	11	15	1.571	2227	15	2016
Rugo Hs	11	14	1.571	3892	14	2016
Iwata H	10	11	1.667	3910	11	2017
Liu J	10	17	2	489	17	2018
Wang Y	10	16	1.667	559	16	2017
Denkert C	9	11	0.9	1109	11	2013

**Figure 4:**
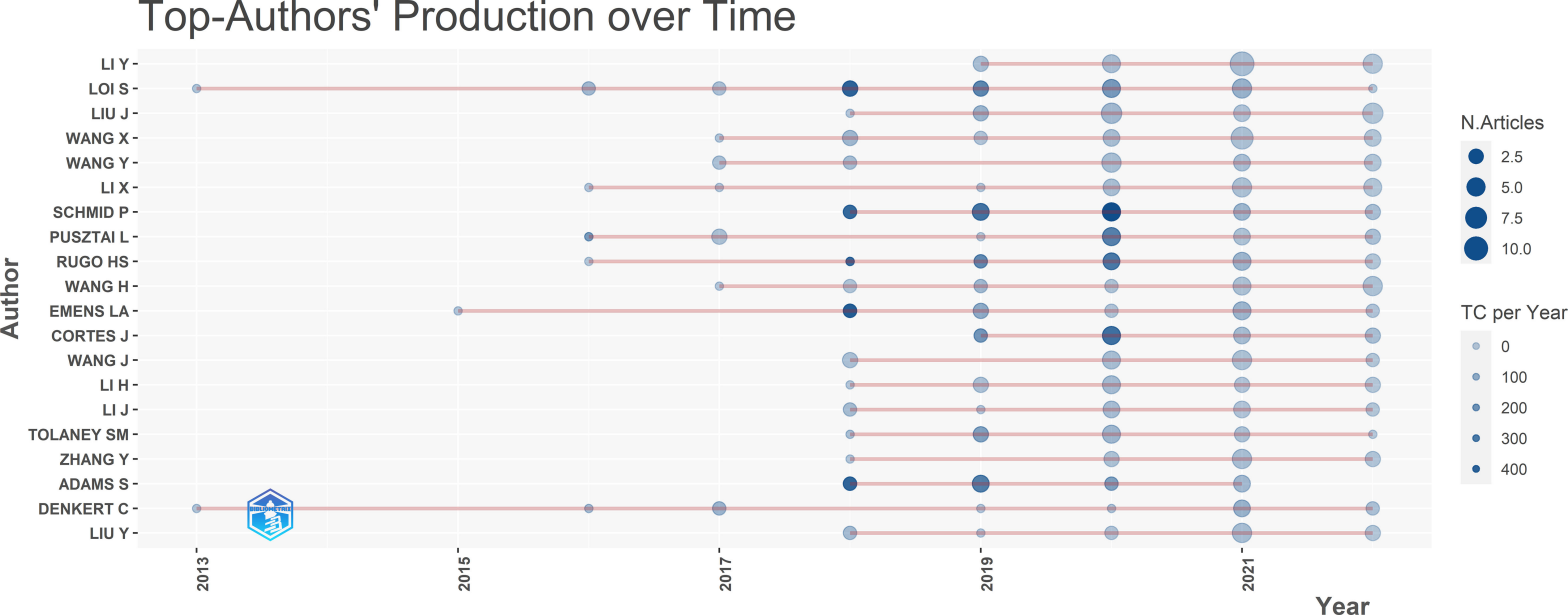
The top 20 authors’ production over time.

The top authors’ production over time is shown in **Figure 4**. It was first published in 2013 by two authors, Loi and Denkert. Most authors began to publish articles related to this field in 2018, and this field has been active for the past 5 years. Schmid has published five articles in total with the highest citation of 481.33 in 2020, among which the title of the one with the highest citations is “Pembrolizumab for Early Triple-Negative Breast Cancer”.

**Figure 5:**
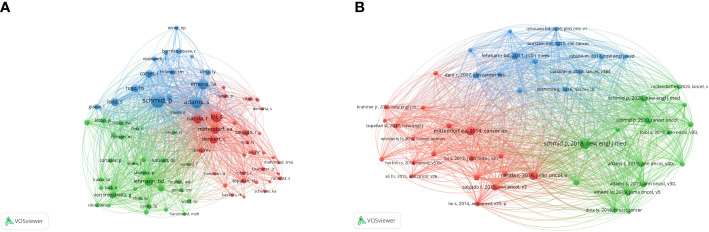
The network visualization of co-citation analysis of authors and references. **(A)** The co-citation analysis of authors. **(B)** The co-citation analysis of reference.

The analysis of the co-citation of authors is shown in **Figure 5A**. A total of 41 authors have more than 50 citations. According to the total link strength, Schmidt had the strongest link strength among other authors (total link strength = 8113), followed by Adams (total link strength = 6022), Emens La (total link strength = 4876), Loi (total link strength = 4671), and Nanda R (total link strength = 3746).

**Figure 6:**
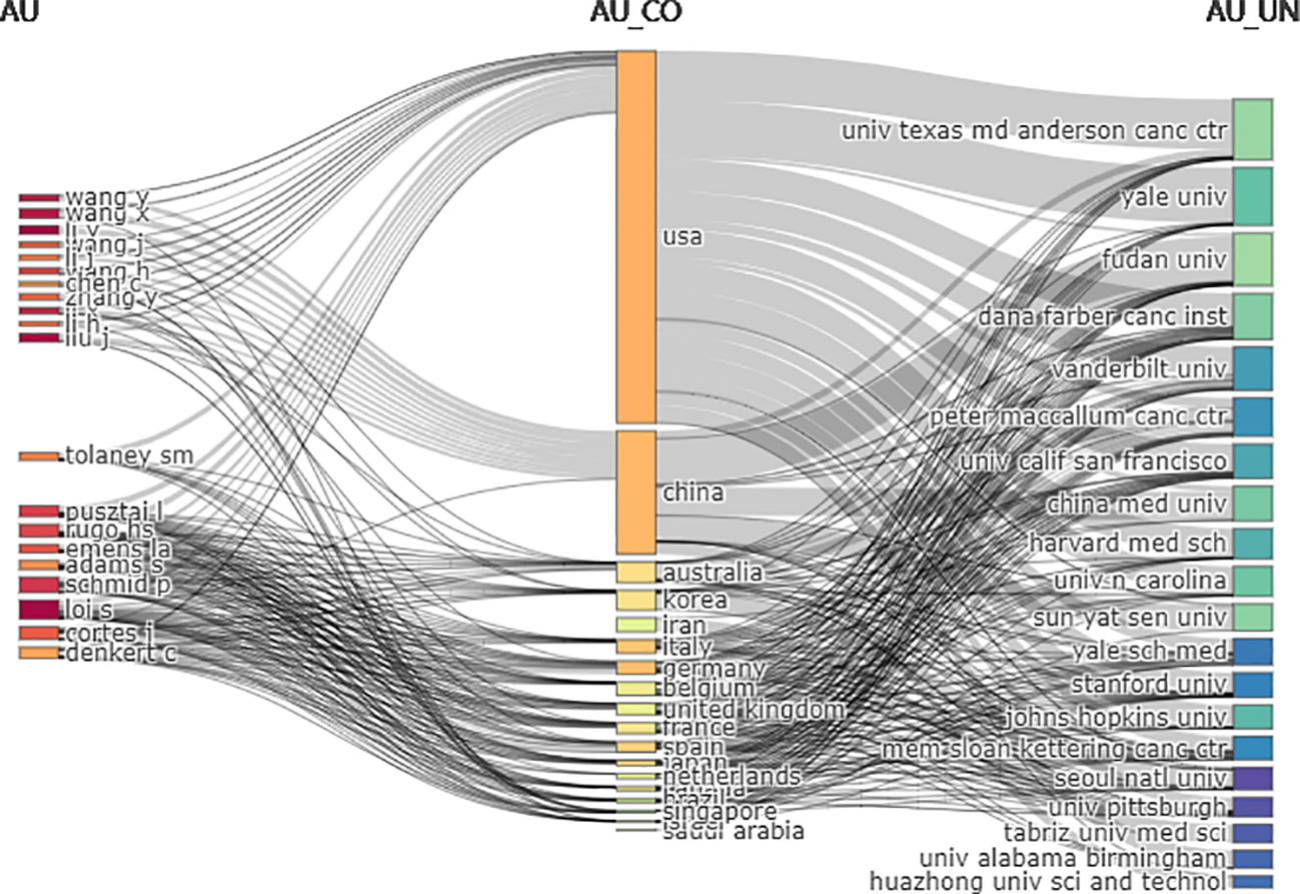
Three-field plot among countries, authors, and affiliations (Left field: authors, middle field: countries, right field: affiliations; number of items each column: 20).

In the three-field plot (**Figure 6**), the authors were set on the left, affiliations were set on the right, and countries were set in the middle. The figure shows the relationship between the country, the author, and the affiliation. The United States had the most connections with authors (20/20) and affiliations (19/20), followed by China (authors = 12/20, affiliations = 15/20). The top contributing affiliations connected with the United States were Dana-Farber Cancer Institute, China Medical University, and Fudan University. The top three authors connected with different countries were Pusztai, Loi, and Cortes.

### 3.5 Reference analysis

We listed the top 10 cited references in the research about anti-PD1/anti-PD-L1 and triple-negative breast cancer in **Table 3**. Highly cited reference could reflect the hot spots of this research, and the content of highly cited reference could show the frontiers of this field. These references were all published between 2014 and 2020. The article “Atezolizumab and Nab-Paclitaxel in Advanced Triple-Negative Breast Cancer” written by Schmid in 2018 had the most total citations (1840 citations) and most total citations per year (368) (18). The article, published in 2016 by Nanda, “Pembrolizumab in Patients With Advanced Triple-negative Breast Cancer: Phase Ib KEYNOTE-012 Study” was cited 1218 times (19). The third-ranked article was “Triple-negative breast cancer: challenges and opportunities of a heterogeneous disease” written by Bianchini (cited 1169 times) (20). Of the top 10 most cited references, there were three written by Schmid, and six clinical trials included one Phase 2 clinical trial, two Phase 1 clinical trials, and three Phase 3 clinical trials.

**Table 3:** The details of the top 10 cited references.

Rank	Title	Corresponding author	Journal	Year	Total Citations	TC per Year(Rank)
1	Atezolizumab and Nab-Paclitaxel in Advanced Triple-Negative Breast Cancer	Schmid P	New England Journal of Medicine	2018	1840	368 (1)
2	Pembrolizumab in Patients With Advanced Triple-Negative Breast Cancer: Phase Ib KEYNOTE-012 Study	Nanda R	Journal of Clinical Oncology	2016	1218	174 (4)
3	Triple-negative breast cancer: challenges and opportunities of a heterogeneous disease	Bianchini G	Nature Reviews Clinical Oncology	2016	1169	167 (5)
4	Pembrolizumab monotherapy for previously treated metastatic triple-negative breast cancer: cohort A of the phase II KEYNOTE-086 study	Adams S	Annals of Oncology	2019	736	184 (3)
5	PD-L1 Expression in Triple-Negative Breast Cancer	Mittendorf Ea	Cancer Immunology Research	2014	681	75.667 (11)
6	Pembrolizumab for Early Triple-Negative Breast Cancer	Schmid P	New England Journal of Medicine	2020	581	193.667 (3)
7	Atezolizumab plus nab-paclitaxel as first-line treatment for unresectable, locally advanced or metastatic triple-negative breast cancer (IMpassion130): updated efficacy results from a randomized, double-blind, placebo-controlled, phase 3 trial	Schmid P	The Lancet Oncology	2020	424	141.333 (6)
8	Molecular alterations in triple-negative breast cancer-the road to new treatment strategies	Denkert C	Lancet	2017	378	63 (16)
9	Avelumab, an anti-PD-L1 antibody, in patients with locally advanced or metastatic breast cancer: a phase 1b JAVELIN Solid Tumor study	Dirix Ly	Breast Cancer Research and Treatment	2018	340	68 (14)
10	A Structured Tumor-Immune Microenvironment in Triple Negative Breast Cancer Revealed by Multiplexed Ion Beam Imaging	Keren L	Cell	2018	340	68 (15)

VOSviewer visualized the co-citation analysis of the reference. The stronger the total link strength, the larger the node is, and more yellow color represents more recent average appearing time (**Figure 5B**). A total of 47 references were analyzed and cited at least 50 times. The article written by Schmid (2018) had the biggest centrality. It was followed by the article written by Mittendorf (2014).

### 3.6 Keyword analysis

**Figure 7:**
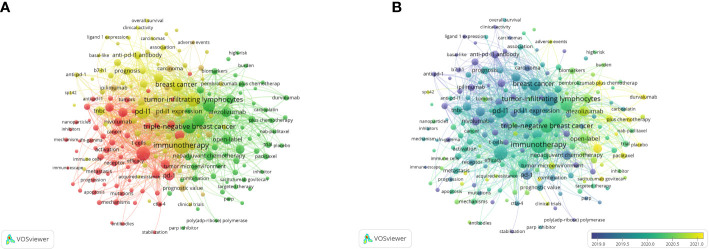
Co-occurrence analysis of keywords. **(A)** The network visualization of keyword co-occurrence analysis. **(B)** The overlay visualization of keyword co-occurrence analysis.

A total of 192 keywords with more than five times of occurrence are shown in **Figure 7A**. The top 5 keywords with the highest link strength were immunotherapy (1396), PD-L1 (964), Triple-negative breast cancer (959), Tumor-infiltrating lymphocytes (937), and Expression (911). These keywords are distinguished by color according to the degree of relevance. It helps to show the all keywords and the focus of recent research. They focus on immunotherapy and chemotherapy for TNBC, prognosis, and expression of PD-L1 and tumor-infiltrating lymphocytes (TILs), and mechanisms and signaling pathways.

In the overlay visualization map (**Figure 7B**). The more yellow the color is, the closer the average occurring time of the keyword is to the present. As time went by, immunotherapy for breast cancer began to be inspired by lung cancer, mainly focusing on triple-negative breast cancer. Study content focuses on the immunotherapeutic mechanisms and signaling pathways of tumors to the immune-related checkpoint inhibitor drugs, the best known of which are atezolizumab and pembrolizumab, which are currently in Phase 3 clinical trials. Recent research has focused on pembrolizumab plus chemotherapy, durvalumab, and double-blind.

Burst keywords indicated the cutting-edge topics in this field over time. **Supplementary Figure 3** shows the top 30 keywords with the strongest citation bursts from 2013 to 2022. These keywords burst citations in 2014–2020. The strength of “poor prognosis” is 6.94, followed by “tumor-infiltrating lymphocyte” (strength = 6.21). The keywords (“proliferation”, “tumor-associated macrophage”, “target”) have been active since 2020, indicating that they are emerging research topics with research potential.

## 4 Discussion

The study searched for anti-PD-1/anti-PD-L1 with TNBC in WoS. The number of scientific publications reflected developments in specific fields based on bibliometrics (21). A total of 851 documents have been searched since the emergence of this research field. The field was first published in 2013, suggesting that TNBC with tumor-infiltrating lymphocytes (TILs) may benefit from immunotherapy (22). From two articles published in 2013 to 280 articles published in 2021, showing a growth trend (annual growth rate: 59.28%). This indicates a gradual increase in the attention to this area. In 2014 the average number of citations reached the peak (85.1), and then it goes down. In 2014, Mittendorf et al. (2014) found that TNBC had higher positivity to express PD-L1 than non-TNBC and the loss of PTEN up-regulated the expression of PD-L1, suggesting the potential role of PD-L1 and the targeting of PI3K pathway could enhance the adaptive immune responses in TNBC. This provides an idea for TNBC immunotherapy (23). There are 16,037 citing documents (self-citing document accounted for 4.75%), and the sum of the times cited was 27,646 (self-cited document accounted for 16.66%). The self-citing rate of citing documents is less than 10%, which means that these citing documents has high credibility, further showing that tracing these citing articles assists interested researchers to have a better understanding of this field in a more comprehensive and objective way. In the top 10 cited references, Schmid published the article “Atezolizumab and Nab-Paclitaxel in Advanced Triple-Negative Breast Cancer” which had the most total citations (1840 citations) and most total citations per year (368). This was a Phase 3 clinical trial about atezolizumab combined with chemotherapy. In patients with metastatic triple-negative breast cancer, atezolizumab plus nab-paclitaxel extended progression-free survival. Of the top 10 cited references, six were clinical trials including one Phase 2 clinical trial, two Phase 1 clinical trials, and three Phase 3 clinical trials. According to this report, triple-negative breast cancer is the focus of current research in this field. The treatment is immunotherapy combined with chemotherapy. TNBC is a highly aggressive and heterogeneous basal-like phenotype tumor (3). Therefore, traditional cytotoxic therapy did not improve survival for triple-negative breast cancer (24).

A total number of 851 documents contributed to this field of research by 65 countries and regions. The United States had the most publications which published 348 papers and accounted for 40.89%. China ranked second with 227 articles published. There were the most citations of publications from the United States, followed by England (n = 3350) and China (n = 3060); 1,586 affiliations contributed to this field. There are six organizations from the United States in the top 10 organizations. This means that the United States has contributed the most to the field. Not only did they publish the majority of studies, but they also received the greatest number of co-citations. Developed countries contribute more to this field than developing countries.

A total of 280 journals were retrieved. The top 10 journals published 173 papers, accounting for 20.33% of all. Approximately 3.04 publications were published per journal on average. The proportion of journals published more than three publications was only 25%. The number of publications in most journals did not exceed the average level, indicating that it is necessary to arouse more related journals’ concern on this topic. Cancers was the most active journals, although it only published 35 articles. The top 10 journals were from the United States and the United Kingdom. All were developed countries. In the top 10 co-cited journals, there were only four journals in the top 10 productive journals. Clinical Cancer Research, Journal for Immunotherapy of Cancer and Annals of Oncology were all in the top five in the number of articles published and co-cited, which indicated that this field has an impact on tumor and immunotherapy. Nine of the top 10 most-produced journals were in the top 10 of the h-index, suggesting that high-impact journals contribute more to the field. “Oncology” accounted for more than half of all documents (514, 60.40%) indicating that cancer subject had the most attention in this field.

These 851 publications were contributed by 5251 authors, including 14 authors of single-authored documents and 5237 authors of multi-authored documents. There were 16 documents written individually. The average number of authors per document was 6.17 and the average number of co-authors documents was 8.72. The top 10 authors published 166 papers, accounting for 19.51% of all. Schmid P and Loi S ranked top in total citations and h-index, Schmid has the largest m-index and Loi has the most publication. A study by Schmid and Loi, published in the Lancet Oncol, showed that atezolizumab plus nab-paclitaxel has a significant clinical effect on PD-L1 positive triple-negative breast cancer after a Phase 3 clinical trial. Moreover, three of the top 10 cited papers were written by Schmid. This indicates that Schmid has the highest academic level in this field, while Loi is the earliest researcher and the most productive author in this field. Both authors have contributed greatly to the research in this field.

The top 5 keywords with the highest link strength were Immunotherapy (1396), PD-L1(964), and Triple-negative breast cancer (959), Tumor-infiltrating lymphocytes (937), and Expression (911). From the perspective of keyword analysis, research in this area focuses on immunotherapy of triple-negative breast cancer and the expression of PD-L1 and tumor-infiltrating lymphocytes. It can be inferred that the transition of the research trend was from the receptor signaling pathway to clinical treatment and prognosis. The best-known checkpoint inhibitor drugs, atezolizumab and pembrolizumab, have complete Phase 3 clinical trials. Recent research has focused on pembrolizumab plus chemotherapy, durvalumab, and double-blind, which indicated that the research on triple-negative breast cancer has taken a step further.

Breast cancer is an immuno-silenced type of cancer, but TNBC has high immunogenicity due to high levels of TILs and higher positive expression of PD-L1 (25, 26). In TNBC, PD-L1 expression has been reported at rates of 19% (23). Therefore, TNBC was more likely to benefit from immunotherapy (23, 27). In 2019, atezolizumab (anti-PD-L1) is the first immune checkpoint inhibitor that received breast cancer approval. A study by Adams et al. on pembrolizumab monotherapy showed a low response rate of 5.3% (28). However, even though PD-1/anti-PD-L1 inhibitor monotherapy has a low efficacy rate, combination chemotherapy was more successful in mTNBC (29).

In 2021, pembrolizumab combined with chemotherapy has been approved by the FDA as a neoadjuvant treatment for high-risk, early-stage, triple-negative breast cancer (TNBC) (30). Atezolizumab or pembrolizumab combined with chemotherapy showed good clinical activity, and pathological complete responses (pCRs) were significantly associated with high levels of PD-L1 (10, 31). In addition, the durvalumab combination in addition to neoadjuvant therapy for early-negative breast cancer is now in Phase 2 trial, and the pCRs with durvalumab were 53.4%. The most common adverse event related to the immune system was thyroid dysfunction (32). Another anti-PD-L1 avelumab with an adjuvant for high-risk TNBC is undergoing a Phase 3 trial (33)

Immune checkpoint inhibitors have had considerable success, but many challenges remain. Some patients had a low response to PD-L1, and some patients developed treatment resistance or toxicity (12). Only a small percentage of patients benefit from immune checkpoint inhibitors, and some patients even experienced serious immune-related adverse events (irAEs) (34). Therefore, some biomarkers are needed to help predict the prognosis of immunosuppressive agents. TILs are currently a prognostic indicator of high pathological complete response rate (pCRs) with neoadjuvant chemotherapy (35). TILs are associated with the survival benefit of TNBC (36). Besides, PD-L1 positive patients also had a better prognosis (37). However, some PD-L1 negative patients also respond to immunotherapy, thus limiting PD-L1 as an exclusive biomarker. PD-L1 positivity has been detected by multiple, distinct companion assays, but their tests have not been compared, standardized, or prospectively validated. Therefore, more validation tests are needed to improve this aspect (38). Predictive biomarkers were being studied extensively. In addition to PD-L1 and TILs, other predictive biomarkers that could be developed were tumor mutational burden (TMB), immune cells, neoantigen, gene signature, and miRNA (39, 40).

## 5 Limitation

Our study has some limitations. We retrieved articles only from the Web of Science Core Collection Database including editions of Science citation index expanded (SCI-Expanded) 2003 to present. No other databases were searched, such as PubMed or Scopus. In addition, the language we retrieved was English. There is certain subjectivity and difference in article screening criteria. It is somewhat subjective to determine and screen articles by referring to other highly cited articles. There was no way to observe the change over time of uncited publications and only self-cited publications. Moreover, we used WoS’s “analyze results” function, bibliometrix package, VOSviewer, and CiteSpace to analyze the included articles, and their calculation methods were different. For example, using VOSviewer’s co-citation analysis of Web of Science data, only the first author of cited documents is included, and other authors are excluded. This will lead to some deviation in the data results. In addition, the time point of our search is July 11, 2022. The search time range of this field was since the documents were published. But since 2022 is not over, data for 2022 were not complete. However, we believe that the results of this analysis can reflect the current research situation in the field and provide some potential research ideas for researchers.

## 6 Conclusion

Triple-negative breast cancer has high immunogenicity due to higher positivity to express TILs and PD-L1. Recently, research on anti-PD-1/anti-PD-L1 for TNBC is rapidly growing. In these papers, the relevant information on anti-PD-1/anti-PD-L-1 immunotherapy for TNBC was analyzed scientifically and quantitatively, revealing the cooperative network of contributing countries, affiliations, and authors, and the current research progress and research hotspots were provided. From molecular mechanisms to clinical treatment and prognosis, the most concerned keywords are “Immunotherapy”, “PD-L1”, “Triple-negative breast cancer”, “Tumor-infiltrating lymphocytes”, and “Expression”, indicating that the research focuses on the immunotherapy of TNBC and the expression of PD-L1 and tumor-infiltrating lymphocytes. Phase III clinical trials of pembrolizumab and atezolizumab plus chemotherapy have been completed. However, biomarkers have limitations in predicting treatment outcomes. Due to the treatment burden of triple-negative breast cancer, there is a huge scope for research in this field to improve prognosis.

## Data availability statement

The raw data supporting the conclusions of this article will be made available by the authors, without undue reservation.

## Author contributions

YKC and LC provided the guidance of the use of these analyzing applications. JW extracted the dataset from Web of Science, performed the statistical analysis and was a major contributor in writing the manuscript. ZJ, JC, HT, JZ, DZ, QY, YL, JC and JZ were involved in the interpretation of the study findings. LC, YL and ZL revised the article critically. All authors contributed to the article and approved the submitted version.
