# Supplementary material for: Global research trends on anti-PD-1/anti-PD-L1 immunotherapy for triple-negative breast cancer: A scientometric analysis
Source: Front Oncol. 2023 Jan 11;12:1002667. doi: 10.3389/fonc.2022.1002667 (PMC9875294; doi:10.3389/fonc.2022.1002667)
Supplement: Supplementary file 1 [file DataSheet_1.docx]

Supplementary Material

# Supplementary Figure 1

#1, TS=("Programmed Cell Death 1 Receptor") OR ("PD-1 Protein") OR ("PD-1 Receptor" OR ("Programmed Cell Death Protein 1") OR ("programmed cell death ligand 1") OR ("PD-L1 protein")OR ("PD-L1 Receptor") OR ("PD 1") OR ("PD L1" OR (“Checkpoint Inhibitors, Immune”)OR ("Immune Checkpoint Blockade") OR ("PD-L1 Inhibitor") OR ("PD-1 Inhibitor") OR ("Programmed Cell Death Protein 1 Inhibitor") OR ("Programmed Death Ligand 1 Inhibitor" OR ("PD-1-PD-L1 Blockade") OR ("anti-PD1") OR ("anti-PD-L1") OR (“PD-1/PD-L1”) OR ( “anti-PD-1/PD-L1”) OR (“Atezolizumab”) OR ("Avelumab") OR ("Durvalumab") OR ("Nivolumab") OR ("Pembrolizumab") OR ("Tislelizumab") OR ("Camrelizumab") OR ("Satralizumab") OR ("Penpulimab") OR ("Toripalimab") OR ("Sintilimab") OR ("Lambrolizumab") OR ("Pidilizumab") OR ("Cemiplimab")

#2, TS=("CTLA-4 Inhibitor") OR ("Cytotoxic T-Lymphocyte-Associated Protein 4 Inhibitor") OR (“CTLA 4”) OR ("anti-CTLA-4") OR ("Abatacept") OR ("Lpilimumab") OR ("Relatlimab") OR ("LAG-3") OR ("anti-LAG-3") OR ("LAG-3 Inhibitor") OR ("lymphocyte activation gene-3") OR ("lymphocyte activation gene-3 Inhibitor")

#3, #1 NOT#2

#4, TS=("Triple-Negative Breast cancer") OR ("Triple-Negative Breast Neoplasm") OR ("ER-Negative PR-Negative HER2-Negative Breast Cancer") OR ("ER Negative PR Negative HER2 Negative Breast Neoplasm")

#5, #3 AND #4

# Supplementary Figure 2

Annual growth of journals


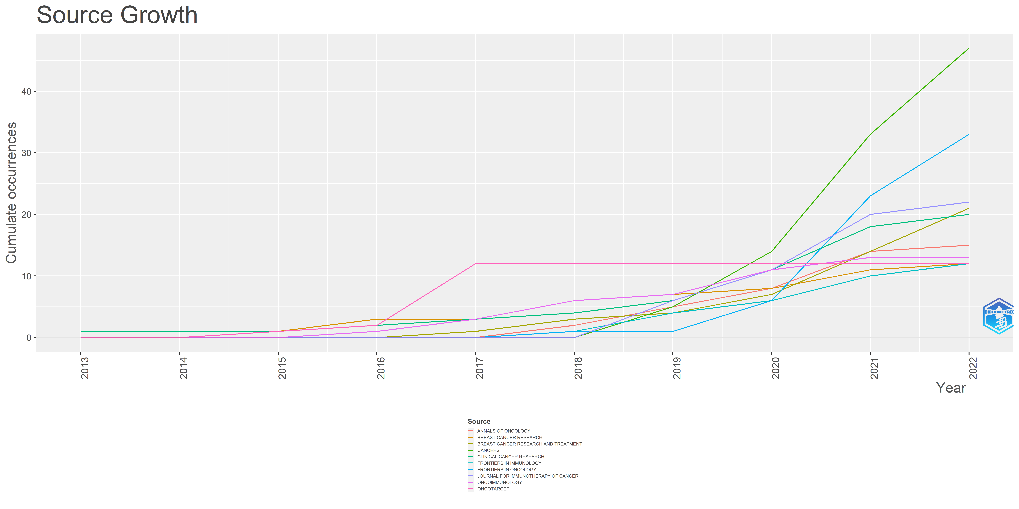


# Supplementary Figure 3

Top 30 keywords with strongest citation bursts


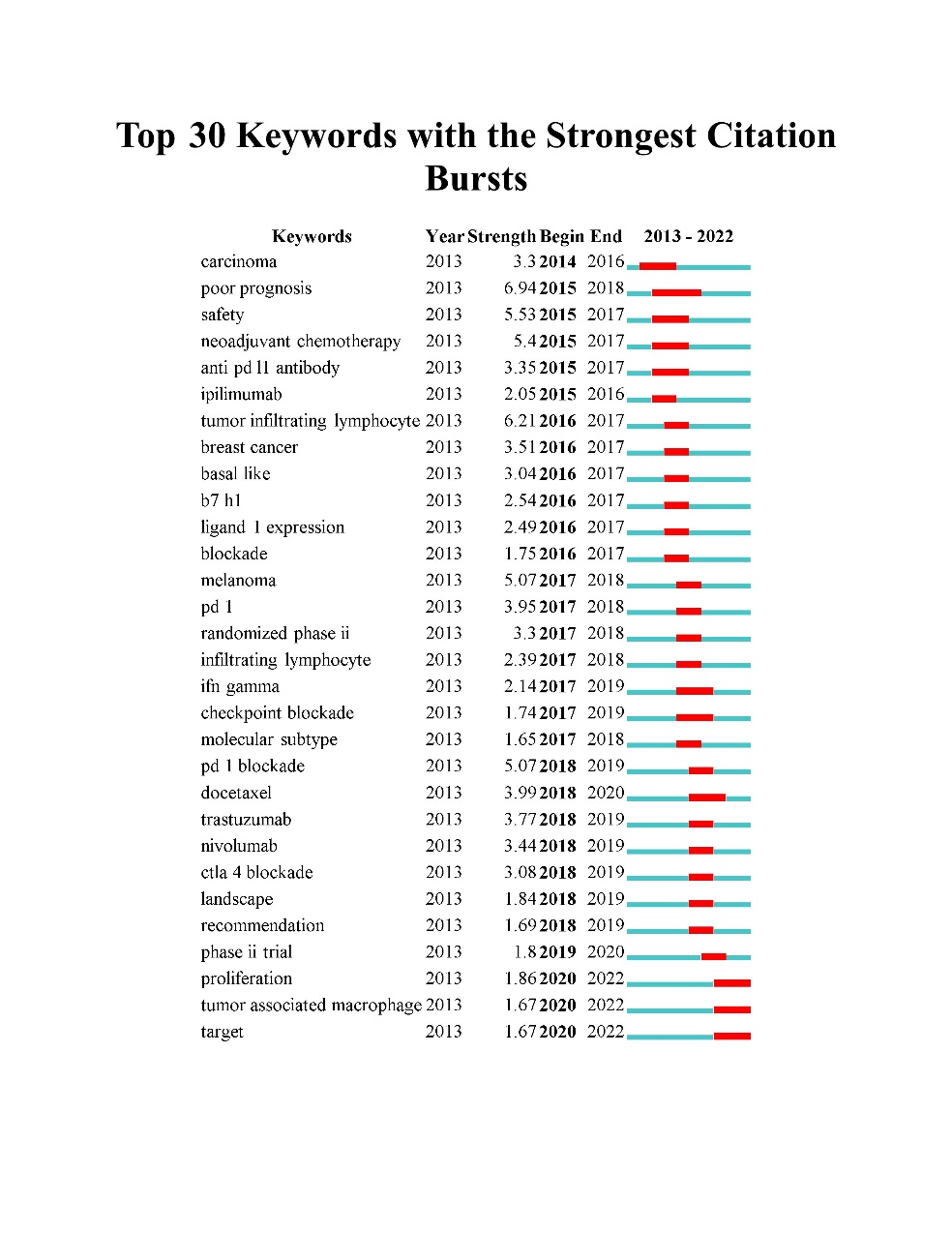


# Supplementary Table 1

The top 10 contributing countries and organizations and top 10 co-cited countries

| Rank | Country | N（%） | Co-cited Country | Citations | Organization(Country) | N(%) |
| --- | --- | --- | --- | --- | --- | --- |
| 1 | USA | 348(40.89%) | USA | 11906 | Harvard university(USA) | 55(6.46%) |
| 2 | China | 227(26.68%) | England | 3350 | University of Texas System(USA) | 44(5.17%) |
| 3 | Italy | 76(8.93%) | China | 3060 | Dana Farber Cancer Institute(USA) | 43(5.05%) |
| 4 | Germany | 63(7.40%) | Italy | 2099 | University of California System(USA) | 41(4.82%) |
| 5 | Japan | 56(6.58%) | Germany | 1158 | Unicancer(France) | 40(4.70%) |
| 6 | Australia | 48(5.64%) | Canada | 913 | Peter MacCallum Cancer Centre(Australia) | 32(3.76%) |
| 7 | UK | 48(5.64%) | Belgium | 632 | Yale University(USA) | 32(3.76%) |
| 8 | France | 42(4.94%) | Australia | 624 | University of London(England) | 30(3.53%) |
| 9 | Belgium | 40(4.70%) | Spain | 544 | UT MD Anderson Cancer Center(USA) | 30(3.53%) |
| 10 | South Korea | 40(4.70%) | Japan | 521 | Udice French Research Universities(France) | 28(3.29%) |

# Supplementary Table 2

# The top 10 active research categories and publishers

| Rank | Research subject category | N(%) | Publisher | N(%) |
| --- | --- | --- | --- | --- |
| 1 | Oncology | 514(60.40%) | Springer Nature | 156(18.33%) |
| 2 | Immunology | 83(9.75%) | Elsevier | 130(15.28%) |
| 3 | Pharmacology Pharmacy | 79(9.28%) | Mdpi | 89(10.46% |
| 4 | Medicine Research Experimental | 64(7.52%) | Frontiers Media Sa | 52(6.11%) |
| 5 | Cell Biology | 62(7.29%) | Wiley | 52(6.11%) |
| 6 | Biochemistry Molecular Biology | 48(5.64%) | Taylor & Francis | 46(5.41%) |
| 7 | Multidisciplinary Sciences | 39(4.58%) | Amer Assoc Cancer Research | 43(5.05%) |
| 8 | Pathology | 36(4.23%) | Lippincott Williams & Wilkins | 24(2.82%) |
| 9 | Obstetrics Gynecology | 35(4.11%) | Bmj Publishing Group | 19(2.23%) |
| 10 | Chemistry Multidisciplinary | 30(3.53%) | NATURE PORTFOLIO | 16(1.88%) |
